# Human neuroimaging studies on the hippocampal CA3 region – integrating evidence for pattern separation and completion

**DOI:** 10.3389/fncel.2014.00064

**Published:** 2014-03-04

**Authors:** Lorena Deuker, Christian F. Doeller, Juergen Fell, Nikolai Axmacher

**Affiliations:** ^1^Department of Epileptology, University of BonnBonn, Germany; ^2^Donders Institute for Brain, Cognition and Behaviour, Radboud University NijmegenNijmegen, Netherlands; ^3^German Center for Neurodegenerative DiseasesBonn, Germany

**Keywords:** high-resolution fMRI, hippocampus, CA3, pattern separation, pattern completion

## Abstract

Human functional magnetic resonance imaging (fMRI) studies have long investigated the hippocampus without differentiating between its subfields, even though theoretical models and rodent studies suggest that subfields support different and potentially even opposite functions. The CA3 region of the hippocampus has been ascribed a pivotal role both in initially forming associations during encoding and in reconstructing a memory representation based on partial cues during retrieval. These functions have been related to pattern separation and pattern completion, respectively. In recent years, studies using high-resolution fMRI in humans have begun to separate different hippocampal subregions and identify the role of the CA3 subregion relative to the other subregions. However, some of these findings have been inconsistent with theoretical models and findings from electrophysiology. In this review, we describe selected recent studies and highlight how their results might help to define different processes and functions that are presumably carried out by the CA3 region, in particular regarding the seemingly opposing functions of pattern separation and pattern completion. We also describe how these subfield-specific processes are related to behavioral, functional and structural alterations in patients with mild cognitive impairment and Alzheimer’s disease. We conclude with discussing limitations of functional imaging and briefly outline possible future developments of the field.

## INTRODUCTION

The hippocampus is something of a lodestone for functional imaging studies in human memory research. Thousands of articles have been published investigating the exact role of the hippocampus (a Pubmed search on October 3rd 2013 for “hippocampus AND human AND memory AND fMRI” returned 2366 results). However, in addition to methodological shortcomings inherent to functional magnetic resonance imaging (fMRI) such as the indirect relationship to neuronal activity and the relatively low signal-to-noise ratio (SNR) and significant susceptibility artifacts in this region (Ojemann et al., 1997; Schacter and Wagner, 1999), many of these studies might also implicitly accept a flawed premise: That the hippocampus is a functional unit, and as such can be imaged and analyzed as a whole.

Everything we know from* in vitro *and animal studies points in the opposite direction. Not only is the hippocampus histologically heterogeneous, but electrophysiological recordings in subfields of the rodent hippocampus suggest a functional dissociation, and circumscribed lesions produce dissociable deficits (Lee and Kesner, 2004a; Lee et al., 2005; Andersen et al., 2006). Some of the hippocampal subfields might even be involved in contrary operations, which could easily lead to null results or opposite conclusions across studies.

Some studies in humans acknowledge a possible functional heterogeneity by considering the anterior and posterior hippocampus differentially (e.g., Ludowig et al., 2008; Chen et al., 2010; Libby et al., 2012; Poppenk et al., 2013), which in rodents maps onto the ventral-to-dorsal axis (Fanselow and Dong, 2010). However, the different subregions of the hippocampus (such as dentate gyrus (DG), and cornu ammonis (CA) regions CA3 and then CA1) extend along the longitudinal axis of the hippocampus and are still collapsed together in these analyses. As this review centers on region CA3, long-axis differentiation (see e.g., Moser and Moser, 1998; Poppenk et al., 2013) will not be further discussed here.

**FIGURE 1:**
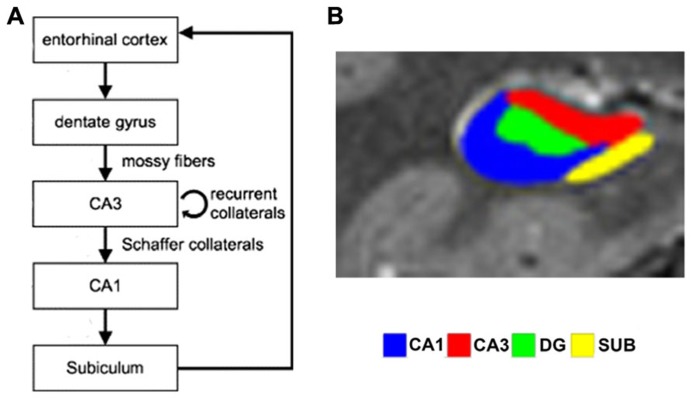
**(A)** Simplified schematic depiction of entorhinal cortex and hippocampal subfields circuitry (modified from Axmacher et al., 2006). In the trisynaptic loop, the dentate gyrus receives input from entorhinal cortex via the perforant path and relays this to CA3 via the mossy fibers. CA3 transfers information to CA1 via Schaffer collaterals, which in turn projects to the subiculum, which routes back to entorhinal cortex. **(B)** Segmented high-resolution fMRI scan showing the different subfields (reproduced with permission from Bonnici et al., 2012).

**FIGURE 2:**
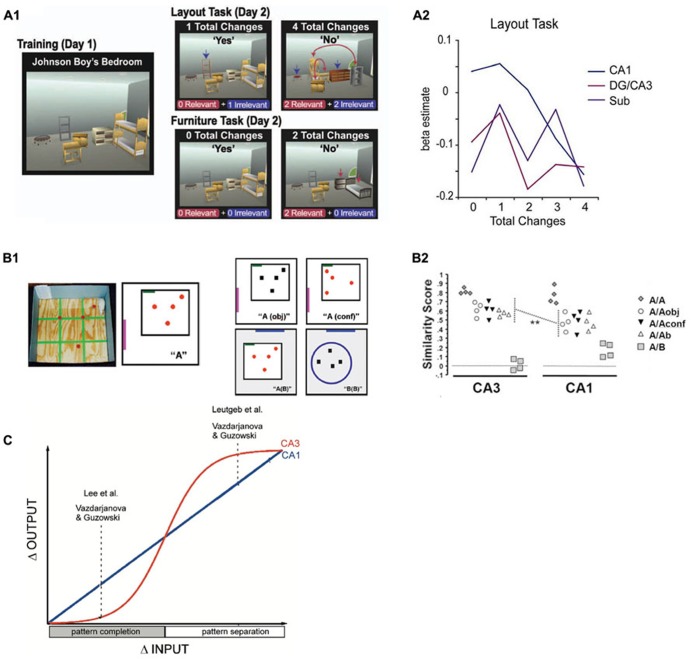
**(A)** Design **(A1)** and results **(A2)** from a functional MRI study in humans (Duncan et al., 2012) in which participants were confronted with different numbers of relevant or irrelevant changes to highly familiar virtual room layouts. Whereas a more linear signal decrease with increasing number of changes was observed in CA1, there was a more sudden, step-like decrease of activity in DG/CA3. Reproduced with permission from Duncan et al. (2012), Copyright © 2011 Wiley Periodicals Inc. **(B)** Design **(B1)** and results **(B2)** from a study in rats (Vazdarjanova and Guzowski, 2004) that investigated the degree of overlap between neuronal ensembles that were active during a training environment **(A)** and a later test environment that was either identical (A/A), increasingly dissimilar (A/Aobj, A/Aconf and A/Ab), or completely different (A/B). Again, CA1 ensembles show linearly decreasing overlap (as captured by a similarity score) with increasing dissimilarity between training and test environment, whereas CA3 ensemble overlap remains fairly high and only drops rapidly in the most dissimilar condition. Reproduced with permission from Vazdarjanova and Guzowski (2004). **(C)** Schematic depiction of the results of three studies in rodents (Guzowski et al., 2004) in which a similar behavior as found in the Duncan et al. (2012) study is described: CA1 output changes linearly with increasing changes in input patterns, while CA3 changes its output more in a sigmoidal fashion (i.e., when a specific threshold is crossed), indicating a transition between pattern completion and pattern separation. Reproduced with permission from Guzowski et al. (2004).

Theoretical models of the function of hippocampal subfields (see **Figure 1** for an overview of the structure) propose that during encoding, CA3 receives sparse, orthogonalized input via the mossy fibers from the DG, an area that in turn receives multimodal input from the entorhinal cortex (ERC; e.g., Lörincz and Buzsáki, 2000; van Strien et al., 2009). Orthogonalization here refers to a theoretical process by which neural patterns are rendered even more dissimilar than they originally were. In CA3, dense recurrent connections are hypothesized to promote rapid conjunctive representations of arbitrary coactive elements – i.e., of elements that are experienced together, but have not been previously linked to each other (Kesner et al., 2008). These anatomical properties are ideally suited to allow CA3 to effectively act as an autoassociative network (Grossberg, 1971; McClelland and Rumelhart, 1985; McNaughton and Morris, 1987). After encoding, CA3 is thought to be able to use a partial or degraded input pattern as a cue to retrieve and complete previously established memory traces, e.g., to retrieve an associated pair from one element of the pair only (O’Reilly and McClelland, 1994; Norman and O’Reilly, 2003; Kesner and Hopkins, 2006), a process called pattern completion (McNaughton and Morris, 1987; McClelland and Goddard, 1996; Colgin et al., 2008). Pattern separation, on the other hand, is the putative computational mechanism which renders partly overlapping neuronal patterns more dissimilar and thus prevents interference and allows novelty detection (Treves and Rolls, 1992; McClelland and Goddard, 1996; O’Reilly and Rudy, 2001). Hence, successful pattern completion depends on previous pattern separation. In other words, a sufficient separation of putatively interfering patterns is a necessary prerequisite for later accurate pattern completion. Rodent studies show that CA3 may promote both pattern completion and pattern separation depending on the degree of similarity or dissimilarity between contexts or learning material (Guzowski et al., 2004; see **Figure 2**). It has been proposed that CA1, in turn, uses the retrieved information from CA3 to compare it to perceptual input from the ERC, acting as a match/mismatch detector between CA3 predictions and EC perceptual input (Jensen and Lisman, 1996; Lee et al., 2004b; Lisman and Grace, 2005; Colgin et al., 2009).

A closer look at the experimental literature on *human* hippocampal subfield functions reveals a gap between these elaborate theoretical and computational models and electrophysiological results on the one hand and what has actually been confirmed in studies in humans on the other. This is mainly because, until recently, the vast majority of fMRI studies collapsed across these different subregions. This lack of discrimination with regard to hippocampal subfields in humans is due to the sheer difficulty to accurately differentiate subfields based on fMRI data in humans. The hippocampus is a relatively small structure, and its subfields are even smaller (on the order of only a few millimeters, with regions CA1, CA2 and CA3 together having an average volume of around 1 cm^3^; see Malykhin et al., 2010). Standard 1.5T or 3T MRI with isotropic voxel sizes of 3–5 mm does not have the resolution to allow for reliable delineation of subfields. Intracranial electroencephalography (EEG) recordings, as they can for example be conducted in epilepsy patients, also cannot contribute because electrode positions usually cannot be determined with the necessary precision – and even if they could, the recorded electric fields are not restricted to the direct vicinity of an electrode, but likely reflect signals from more than one subfield. Microelectrodes have recently been employed in addition to clinically used macroelectrodes, and they record from small areas; often, single-cell activity can be identified (e.g., Suthana and Fried, 2012). However, the position of these microelectrodes often cannot be accurately ascribed to a specific subfield.

In the last decade, more powerful MR scanners with strong magnetic fields of 7T and beyond have become available. Together with scanning parameters specifically optimized for imaging medial temporal lobe structures (Weiskopf et al., 2006; Bakker et al., 2008; Doeller et al., 2008, 2010; Ekstrom et al., 2009; Bonnici et al., 2012), notorious for signal dropouts and low SNR, some studies have successfully extracted functional activity from hippocampal subfields. Another promising approach is the collection of multiple high-resolution structural scans and averaging them together for better signal quality (Bonnici et al., 2012; Newmark et al., 2013). Segmentation is most often done manually, based on specific landmarks (e.g., as described in Duvernoy, 2005) and can become difficult in the head and tail of the hippocampus. Therefore, only the body is segmented in many studies, while others do segmentation along the entire length. This can lead to differences across studies with regard to volumetry and to a skewed representation of subfields: the proportion of DG volume is lower in anterior than in posterior hippocampus whereas the proportion of CA1, CA2 and CA3 volume is higher in anterior than posterior hippocampus (Malykhin et al., 2010; Poppenk et al., 2013). Coregistration of subfields across participants is still a challenge, even though toolboxes have become available in the last years (e.g., ROI-AL, http://darwin.bio.uci.edu/simcestark/roial; see Yassa and Stark, 2009 for a review). Also, separation of CA3 from DG remains difficult and the two subregions are often collapsed, even though there is good reason to assume that they support different functions. However, some studies report reliable separation of CA3 from DG (Bonnici et al., 2012; Wisse et al., 2012). Recently, an initiative to standardize the procedures for subfield delineation has been founded, which will hopefully move the field towards studies with more easily comparable results (www.hippocampalsubfields.com).

In this review, we first summarize selected fMRI studies that aimed to differentiate between hippocampal subregions in healthy participants. We discuss how these studies contribute to testing the theoretical models outlined above in terms of the processes thought to be performed by the CA3 subregion. We focus on CA3 and mention results on other subregions only where necessary for understanding the specific role of CA3. We then consider investigations in patient populations with subfield-specific damage. Finally, we conclude by discussing future developments which could potentially allow us to investigate the CA3 region in greater detail – possibly even resolving different subparts of this region. **Table 1** provides an overview of the main findings in selected recent studies and the subfields that have been delineated in them.

**Table 1:** Overview of selected publications which have investigated the function of CA3 and other MTL and hippocampal subfields in humans.

Paper	ERC	PRC	PHC	CA1	CA2	CA3	DG	SUB	Results (CA3)
Bakker et al. (2008)									Pattern separation during encoding
Bonnici et al. (2012)									Pattern completion
Carr et al. (2010b)									Subsequent memory only for late recall
Chen et al. (2011)									Correct vs. incorrect retrieval
Dudukovic et al. (2011)									Pattern completion
Duncan et al. (2012)									Sigmoidal dependence on similarity
Eldridge et al. (2005)									Encoding > retrieval
Lacy et al. (2011)									Sigmoidal dependence on similarity
Mueller et al. (2007)									Preserved function in AD
Mueller et al. (2010)									Preserved function in MCI and AD
Newmark et al. (2013)									Pattern completion
Olsen et al. (2009)									Activation during DMS sample and delay
Schapiro et al. (2012)									Increased similarity via temporal proximity
Suthana et al. (2011)									Encoding > retrieval
Wisse et al. (2012)									Reliable manual segmentation
Yassa et al. (2010b)									Hyperactivity during pattern separation in aMCI
Zeineh et al. (2003)									Encoding > retrieval

*ERC, entorhinal cortex; PRC, perirhinal cortex; PHC, parahippocampal cortex; CA1–CA3, cornu ammonis subregions 1–3, respectively; DG, dentate gyrus; SUB, subiculum. Gray boxes indicate the subfields that have been segmented in a given study; most studies combined subfields CA2, CA3, and dentate gyrus as indicated by gray boxes stretching several columns. The last column summarizes the main finding with regard to CA3 function.*

## FUNCTIONAL IMAGING IN HEALTHY PARTICIPANTS

As mentioned above, a wealth of theories about the role of hippocampal subfields for memory exist (Marr, 1971; McNaughton and Morris, 1987; Treves and Rolls, 1992; McClelland and Goddard, 1996), in particular on the role of CA3. The most prevalent assumptions can be summarized as follows: (1) CA3 is important for memory encoding, (2) during encoding, CA3 processes orthogonalized input that likely is a result of pattern separation processes in DG and further supports pattern separation of these relatively dissimilar inputs, (3) CA3 promotes binding of dissociated elements, and during retrieval uses parts of a pattern to retrieve the entire pattern (pattern completion). In the following, we consider how recent studies in humans have contributed to shedding more light on these predictions.

### CA3 IS IMPORTANT FOR MEMORY ENCODING

It has long been established that the hippocampus per se is critically important for memory formation (Scoville and Milner, 1957). But does subregion CA3 play a special role in encoding? As has been reviewed before (Carr et al., 2010a), some high-resolution studies on the hippocampus have found evidence that CA3 is more involved in encoding than retrieval (Zeineh et al., 2003; Suthana et al., 2011). Alternatively, it was shown that CA3 is more involved in encoding than other hippocampal subregions are. For example, a subsequent memory paradigm revealed that CA2/3/DG but not subiculum (SUB) predicted later memory success for pairs of line-drawing objects (Eldridge et al., 2005). In a delayed matching to sample (DMS) study, increased CA2/3/DG activity was observed during the sample and early delay phase (i.e., encoding related phases), whereas CA1 was more active during the delay and the test phase (Olsen et al., 2009). A more recent study found a subsequent memory effect in DG/CA2/3 that could not be detected in CA1 (Carr et al., 2013). However, some studies also find that encoding and novelty detection are not restricted to CA3 (Chen et al., 2011; Duncan et al., 2012).

It should be noted that in these studies, the fMRI signal from a combined region of CA2, CA3 and DG (CA2/3/DG) was analyzed, which limits interpretation. Also, the time between encoding and retrieval differs vastly, from 30 s (Olsen et al., 2009) to a week (Carr et al., 2010b). These differences should be considered in future studies because with very short time periods (e.g., in DMS tasks), one most likely investigates working memory maintenance, while only with longer periods, long-term memory processes are actually considered. Moreover, the involvement of CA2/3/DG appears to be related to the long-term stability of memories. This issue is illustrated in a study by Carr et al. (2010b) in which CA2/3/DG reflected successful encoding only for those items for which the memory lasted not only transiently (10 min) but also permanently (1 week).

### CA3 SUPPORTS PATTERN SEPARATION OF DISSIMILAR INPUTS DURING ENCODING

Several fMRI studies have investigated the role of the CA3 region related to pattern separation and completion during memory encoding, for example two studies from Craig Stark’s lab (Bakker et al., 2008; Lacy et al., 2011). In the first study (Bakker et al., 2008), the experimental task contained three types of trials: new items, repeated items or lures (new items which were very similar to already shown items). The authors then looked for repetition effects, i.e., attenuation of blood oxygen level dependent (BOLD) responses for novel, lure and repeated stimuli (Grill-Spector et al., 2006). The logic behind this is that lures are “intermediate” between novel items and repeated (identical) items and the authors used the presence or absence of BOLD attenuation during lure trials as an indication of whether the brain treated the lure stimuli more like novel or more like familiar stimuli. CA1 exhibited a tendency for pattern completion by showing repetition suppression for lures suggesting they were processed more like an already encountered object rather than a novel stimulus. DG/CA3 in contrast did not show repetition suppression for lures, which is indicative of pattern separation: the lure was treated more like a completely new object. In a follow-up study (Lacy et al., 2011), a linear modulation of repetition suppression in CA1 for increasingly dissimilar items (repeat, high similarity lure, low similarity lure, and new items) was found. In DG/CA3, attenuation of the BOLD response was more step-like: Only true repeat items were associated with repetition suppression, while neither high nor low similarity lures led to an attenuation of the BOLD response. These results are also consistent with a study by Duncan et al. (2012), in which participants were exposed to room layouts with different numbers of changes relative to previously learned rooms. CA1 was the only hippocampal subregion in which activation was linearly modulated by the number of changes. By contrast, DG/CA3 showed more of an abrupt, binary response, which might reflect a switch from pattern completion to pattern separation. This is, however, not discussed in this study, even though it complements a similar study in rodents using an immediate early gene brain imaging approach (Vazdarjanova and Guzowski, 2004) that investigated the degree of overlap of neuronal ensembles between learning and test environments. In this study, rats were familiarized with an environment and a set of objects. After 20 min, they were placed in environments that ranged from being very similar to very dissimilar to the original environment and set of objects. Only in the very dissimilar environment, the degree of overlap between neuronal ensembles decreased abruptly. This step-like response pattern was not seen in CA1 and it suggests that depending on the input dissimilarity, CA3 switches from pattern completion to pattern separation. However, another study in rodents reports that sudden, step-like like remapping of place cells in incrementally dissimilar environments can also occur in CA1 (Wills et al., 2005).

That CA3 should be involved in pattern separation-like processes seems to be at odds with the notion that this subfield performs pattern completion (McNaughton and Morris, 1987; McClelland and Goddard, 1996; Colgin et al., 2008). One explanation for this apparent divergence of results from theoretical predictions could be that during the different stages of learning (e.g., encoding versus retrieval), the same region might support different processes, i.e., pattern separation during encoding and pattern completion during retrieval, as has been discussed before (Hunsaker and Kesner, 2013). Furthermore, it is not clear whether the BOLD response in a region reflects more synaptic input (e.g., from ERC) or processing in this region per se; see below in the discussion on the limitations of human subfield imaging studies.

In another high resolution fMRI study (Newmark et al., 2013), a DMS task was used. Participants were exposed to two face stimuli that either had overlapping features (same identity, different facial expression) or had non-overlapping features (different identity, different facial expression). Higher activity during working memory encoding in trials with overlapping samples was found in the right CA3/DG and in bilateral CA1. Higher activity for overlapping samples during the 8 s of maintenance was found in right CA1 and SUB only. Similarly, Dudukovic et al. (2011) report more activation in CA1 and CA3/DG during the test phase in a DMS task when the tested item matched the sample than when the tested item did not match the sample. They interpret this “match enhancement” as an indication for pattern completion.

The studies described thus far used classical univariate approaches to data analysis, investigating whether BOLD activity systematically differs between conditions, usually modeled within the general linear model (GLM) framework. However, these approaches are limited in elucidating the functional role of different hippocampal subfields, because many theoretical models conceptualize the subfields in terms of information content rather than activation level. Therefore, multivariate pattern analysis (MVPA) approaches may not only uncover differences that would not be detected in classical GLMs, but may be conceptually better suited to address predictions from these models. Bonnici et al. (2012) performed pattern classification analyses on voxels belonging to different hippocampal subfields while participants viewed scenes that were either purely scene A or scene B or ambiguous scenes which were morphed continuously between scenes A and B. Participants had to decide whether the presented scene was scene A or scene B, which was easy for the pure scenes (with which participants were presented during the first part of the study), but was more difficult or completely arbitrary for the morphed scenes, with which participants were confronted during the second part of the study. In this study, in addition to CA1 and SUB, CA3 and DG were delineated as separate subfields. Interestingly, classifier accuracy was better when classifying subjects’ responses to morphed scenes (i.e., trials with high perceptual ambiguity) than to “pure scenes”, and in these ambiguous trials, CA1 and CA3 had better classification accuracy than DG and SUB. Higher classification accuracy for ambiguous stimuli was interpreted by the authors as evidence for a pattern completion process due to the stronger need to retrieve internal representations in these trials. This result supports the idea that CA1, together with information from CA3, acts as a mismatch detector between stored information and perceptual input from the ERC (Fries, 2009).

In summary, recent studies have provided evidence for both pattern separation and pattern completion-like processes in hippocampal subfield CA3 during working and long-term memory operations, even though these results are not always specific for CA3 but are sometimes also reported for CA1. Which of the two processes is observed in CA3 likely depends on a variety of factors such as the degree of similarity or dissimilarity of inputs and whether the stimulus material in general is novel (as in Bakker et al., 2008; Lacy et al., 2011) or highly familiar (as, for example, in Duncan et al., 2012; Newmark et al., 2013), because different processes are likely involved in processing these two types of stimuli (for example, encoding versus retrieval, as discussed above).

### CA3 PROMOTES BINDING DURING ENCODING AND PATTERN COMPLETION DURING RETRIEVAL

A key function that is attributed to CA3 is the binding of previously not associated elements. An MVPA approach was used by Schapiro et al. (2012) to investigate how representational similarity (i.e., pattern similarity in fMRI scans) for unfamiliar, unrelated fractal pictures would change if they were presented repeatedly in a temporally structured manner. First, they presented abstract fractal pictures in random order. Then, the fractals were presented again with some fractals forming pairs in which one always followed the other (strong pairs) or followed the other in a third of all cases (weak pairs). Finally, fractals were shown in a random order again, and representational similarity independent of temporal proximity between the pairs was compared between the initial and the final scanning. Similarity for strong pairs relative to non-pairs and weak pairs increased in SUB, CA1 and combined CA2/CA3/DG, thereby showing that a temporal association between items increases the similarity of their representations, but only CA2/3/DG did so in a forward-looking, predictive manner (the first of a pair leading to reinstatement of the second part of the pair, but not the other way around). This study provides support for both the notion that CA3 is involved in forming arbitrary associations (e.g., between previously unrelated fractals), but also suggests that after encoding, CA3 uses parts of the newly formed association to retrieve the complete pattern, i.e., pattern completion.

In a similar vein as Schapiro et al. (2012), Chen et al. (2011) let participants study house-face pairs and during recall presented them with only one part of the pair. Participants had to covertly recall the associated house or face. After 7.5 s, participants were presented with a probe which was either a match (the correct partner of the pair) or a foil (belonging to another pair). Activation in an anatomical CA2/CA3/DG ROI was higher during the covert retrieval phase if participants subsequently responded correctly to the probe, which would be consistent with CA3 retrieving the associated pair.

Investigating how hippocampal subfields react to pairs of stimuli is a promising approach. On the one hand, this line of research allows one to test the hypothesis that the CA3 region promotes rapid binding of disparate elements. MVPA approaches might in future be used to track the “learning” process of such associative pairing (i.e., how similarity changes over the course of a learning process). On the other hand, paired associative studies also allow testing the assumption that CA3 uses parts of a memory trace to reinstate or retrieve the complete trace. In pair-association studies, it would be especially interesting to separate DG from CA3 because these two regions in such tasks likely perform different or even opposite operations, which may make it difficult to observe significant findings for the regions in the first place.

## STRUCTURAL AND FUNCTIONAL ALTERATIONS IN ALZHEIMER’S DISEASE AND MILD COGNITIVE IMPAIRMENT

Investigations into the function of hippocampal subfields in humans may also benefit from studies in patient populations in whom these functions are disturbed. Accordingly, the link between changes in hippocampal subfields and psychiatric and neurological diseases has been intensively investigated (for a review, see Small et al., 2011). Of special interest here is Alzheimer’s disease (AD), not only because it is primarily a memory disorder, but also because of its high prevalence, making it a major medical issue in an increasingly aging population. Mild cognitive impairment (MCI) has also been the target of many studies because MCI patients often progress to AD (Petersen et al., 1999), especially in case of the amnesic subtype which is characterized by subjective and objective unusual impairment in memory in the presence of preserved general cognitive abilities and function in daily activities (Petersen, 2004). Studies on amnesic MCI (aMCI) patients therefore offer the possibility to detect early symptoms and alterations in the memory system.

Healthy aging and AD are associated with different morphological changes in the hippocampal formation: In AD, volume loss occurs rather in ERC, CA1 and SUB than in CA3 or DG (Mueller et al., 2007, 2010). By contrast, in healthy aged as compared to healthy young humans the perforant pathway which connects ERC and hippocampus is specifically degraded, and the degree of fiber loss is correlated with the degree of behavioral memory deficits (Yassa et al., 2010a). Furthermore, perforant path degradation was associated with diminished pattern separation abilities on a behavioral level and to a lack of pattern separation-like activity in DG/CA3 on a functional MRI level (Yassa et al., 2011).

It has been suggested that one of the first behavioral deficits in patients with aMCI is a reduced ability to separate patterns (i.e., to recognize differences between very similar events), and that this basic deficit accounts for many of the amnesic symptoms (Yassa et al., 2010b). To address this question, Yassa et al. (2010b) investigated patients with aMCI. In addition to differences in volume and shape of hippocampal subfields in patients, they found reduced activity in the ERC during an encoding task that employed highly similar “lure” items and thus required pattern separation for successful performance. They found behavioral deficits in pattern separation compared to healthy controls and hyperexcitability of the CA3/DG region during the task. Interestingly, this corresponds well to results from Wilson et al. (2005) who reported increased firing in CA3 place cells in healthy aged rats. This hyperactivity could be a sign for a computational shift from pattern separation to pattern completion (Yassa et al., 2010b). In contrast to Mueller et al. (2010), the study by Yassa et al. (2010b) also found smaller CA3/DG volumes in aMCI patients. One possible explanation for these conflicting findings might relate to the different segmentation methods used in the studies, especially with regard to the longitudinal extent in which subfields were delineated.

A pattern separation deficit in aMCI, associated with decreased volume and hyperexcitability in CA3/DG, seems to be at odds with the notion that CA3/DG is relatively spared in patients with AD (assuming that some of the aMCI patients are on the way to developing the disease). Differences in segmentation methods could be one explanation for the different conclusions. In addition, patients with aMCI also exhibit abnormal activity in the ERC, which could lead to altered downstream DG and CA3 function even in the absence of structural alterations in CA3 itself (Yassa et al., 2010b).

Clearly, more research is needed to investigate the specific behavioral deficits of patients with aMCI and relate them to structural and functional changes in CA3. Paradigms asking participants to differentiate between highly similar stimulus material provide a good model for the investigation of pattern separation processes, but they should be complemented by experiments which require participants to retrieve paired associates, which is another major function ascribed to CA3. The field will also benefit from studies that look at healthy young populations who have a genetic risk for developing AD such as carriers of the epsilon4 subtype of the apolipoprotein E gene (Corder et al., 1993).

## OUTLOOK – NEW METHODS

Taken together, recent high-resolution MRI and fMRI studies have provided support for theoretical models with regard to pattern completion and pattern separation functions of CA3, its key role in forming new associations and relevance for retrieval of complete patterns based on only some parts of a memory trace. It has become clear from the studies described above that it is essential to use well designed studies that control factors such as incidental versus instructed encoding, whether novel or highly familiar stimuli are used and whether working memory or long-term memory processes are investigated.

It also seems to be important to develop methods for reliable separation of CA3 from DG. Combining these two subfields may lead to conflicting results. Also, the extent of subfield delineation along the longitudinal axis of the hippocampus should be more consistent across studies, especially because the relative proportion of CA3 and DG volume might be affected by the different portions of the hippocampus that are included (Poppenk et al., 2013).

Despite the many interesting findings that research on hippocampal subfields in humans has yielded so far, interpretation should always be careful. Even when stepping from theoretical models to rodent studies, some discrepancies can be observed between model and data, which are likely due to both methodological difficulties as well as the challenge of operationalizing specific memory processes. In humans, especially with fMRI imaging, these problems increase further. The BOLD response is a coarse signal when compared to single cell activity, both in time and in space. It is not possible to unequivocally attribute the BOLD signal to input, output or local processes within a subregion (Buzsáki et al., 2007; Logothetis, 2008), which will not be fixed even by higher resolution scanning, and signal “spill-over” between regions also has to be expected, even if difficult-to-delineate subfield such as CA3 and DG should one day be successfully and routinely segmented.

An integrative approach is required to bring together data from different modalities (such as animal research, intracranial EEG, lesion studies, volumetry studies in patient populations and high resolution functional MRI) with refined theoretical models and well-conceived standard paradigms. We expect that in the next decade, availability of high-field MRI scanners and the development of new scanning protocols will allow vastly improved delineation of subfields. Studies using 7T report resolutions as fine as in 0.8 mm, optimized for resolution of individual cell layers, in fMRI (De Martino et al., 2013) and temporal resolution may also be decreased to 700 ms for fMRI (Smith et al., 2013) or even 50 ms with Generalized iNverse imaging (GiN; Boyacioǧlu, 2012), albeit at the expense of spatial specificity or SNR. New protocols might also allow better structural scanning in 1.5T or 3T scanners, which is especially important for good localization in patients with intracranial electrodes who cannot be scanned at higher magnetic field strengths. This might allow us to draw conclusions about specific subfields in these valuable participants as well. Also, multivariate approaches are an exciting new possibility to investigate hippocampal subfields, because they can assess information content rather than BOLD activity level and might allow for the detection of differences that would be missed with classical univariate methods. This method might also be used to track the emergence of associations between the two parts of a pair and test whether, during retrieval, parts of a pair induce reinstatement of the complete trace, which is one of the main processes attributed to CA3. For elucidating the exact role of CA3 in encoding and retrieval and whether CA3 supports pattern separation, pattern completion or both in different parts of the learning process, it is critical to carefully choose a paradigm that permits investigating the purported functions in detail and to integrate results from different research techniques and questions. Importantly, paradigms which approach the goal of process purity as close as possible should be applied. As Hunsaker and Kesner (2013) argue, in many cases encoding and retrieval processes are mingled due to the use of everyday objects, representations of which are likely already stored and will be retrieved at the time of experimental encoding, thereby further increasing the difficulty of dissociating pattern separation and pattern completion. This problem might be circumvented by using abstract, never-before-seen objects during encoding for which no prior associations have been formed.

Taken together, improved scanning and stronger experimental control might, in the future, lead to better understanding, more accurate diagnosis and even targeted treatment of memory disorders.

## AUTHOR CONTRIBUTIONS

Lorena Deuker, Christian F. Doeller, Juergen Fell, and Nikolai Axmacher wrote the manuscript.

## Conflict of Interest Statement

The authors declare that the research was conducted in the absence of any commercial or financial relationships that could be construed as a potential conflict of interest.

## Abbreviations

AD: Alzheimer’s disease
aMCI: amnesic mild cognitive impairment
BOLD: blood oxygen level dependent
CA: ornu ammonis
DG: dentate gyrus
DMS: delayed matching-to-sample
EEG: electroencephalography
ERC: entorhinal cortex
fMRI: functional magnetic resonance imaging
GLM: general linear model
MCI: mild cognitive impairment
MTL: medial temporal lobe
MVPA: multi-voxel pattern analysis
PHC: parahippocampal cortex
PRC: perirhinal cortex
SNR: signal-to-noise ratio
SUB: subiculum

## References

[B1] Andersen P. Morris R. Amaral D. Bliss T O’Keefe J. (2006). *The Hippocampus Book*. New York: Oxford University Press 10.1093/acprof:oso/9780195100273.001.0001

[B2] Axmacher N. Mormann F. Fernández G. Elger C. E. Fell J. (2006). Memory formation by neuronal synchronization. *Brain Res. Rev.* 52 170–182 10.1016/j.brainresrev.2006.01.007 16545463

[B3] Bakker A. Kirwan C. B. Miller M Stark C. E. L. (2008). Pattern separation in the human hippocampal CA3 and dentate gyrus. *Science* 319 1640–1642 10.1126/science.1152882 18356518 PMC2829853

[B4] Bonnici H. M. Chadwick M. J. Kumaran D. Hassabis D. Weiskopf N. Maguire E. A. (2012). Multi-voxel pattern analysis in human hippocampal subfields. *Front. Hum. Neurosci. * 6:290 10.3389/fnhum.2012.00290 PMC3474998 23087638

[B5] Boyacioǧlu R. Barth M. (2012). Generalized iNverse imaging (GIN): ultrafast fMRI with physiological noise correction. *Magn. Reson. Med*. 70 962–971 10.1002/mrm.24528 23097342

[B6] Buzsáki G. Kaila K. Raichle M. (2007). Inhibition and brain work. *Neuron* 56 771–783 10.1016/j.neuron.2007.11.008 18054855 PMC2266612

[B7] Carr V. A. Engel S. A. Knowlton B. J. (2013). Top-down modulation of hippocampal encoding activity as measured by high-resolution functional MRI. *Neuropsychologia* 51 1829–1837 10.1016/j.neuropsychologia.2013.06.026 23838003 PMC3829781

[B8] Carr V. A. Rissman J. Wagner A. D. (2010a). Imaging the human medial temporal lobe with high-resolution fMRI. *Neuron* 65 298–308 10.1016/j.neuron.2009.12.022 20159444 PMC2844113

[B9] Carr V. A. Viskontas I. V. Engel S. A. Knowlton B. J. (2010b). Neural activity in the hippocampus and perirhinal cortex during encoding is associated with the durability of episodic memory. *J. Cogn. Neurosci.* 22 2652–2662 10.1162/jocn.2009.21381 19925190

[B10] Chen J. Olsen R. K. Preston A. R. Glover G. H. Wagner A. D. (2011). Associative retrieval processes in the human medial temporal lobe: hippocampal retrieval success and CA1 mismatch detection. *Learn. Mem.* 18 523–528 10.1101/lm.2135211 21775513 PMC3256570

[B11] Chen K. H. Chuah L. Y. Sim S. K. Chee M. W. (2010). Hippocampal region-specific contributions to memory performance in normal elderly. *Brain Cogn.* 72 400–407 10.1016/j.bandc.2009.11.007 20044193

[B12] Colgin L. L. Denninger T. Fyhn M. Hafting T. Bonnevie T. Jensen O. (2009). Frequency of gamma oscillations routes flow of information in the hippocampus. *Nature* 462 353–357 10.1038/nature08573 19924214

[B13] Colgin L. L. Moser E. I. Moser M.-B. (2008). Understanding memory through hippocampal remapping. *Trends Neurosci.* 31 469–477 10.1016/j.tins.2008.06.008 18687478

[B14] Corder E. H. Saunders A. M. Strittmatter W. J. Schmechel D. E. Gaskell P. C. Small G. W. (1993). Gene dose of apolipoprotein E type 4 allele and the risk of Alzheimer’s disease in late onset families. *Science* 261 921–923 10.1126/science.8346443 8346443

[B15] De Martino F. Zimmermann J. Muckli L. Ugurbil K. Yacoub E. Goebel R. (2013). Cortical depth dependent functional responses in humans at 7T: improved specificity with 3D GRASE. *PLoS ONE * 8:e60514 10.1371/journal.pone.0060514 PMC3606277 23533682

[B16] Doeller C. F. Barry C. Burgess N. (2010). Evidence for grid cells in a human memory network. *Nature* 463 657–661 10.1038/nature08704 20090680 PMC3173857

[B17] Doeller C. F. King J. A. Burgess N. (2008). Parallel striatal and hippocampal systems for landmarks and boundaries in spatial memory. *Proc. Natl. Acad. Sci. U.S.A.* 105 5915–5920 10.1073/pnas.0801489105 18408152 PMC2311337

[B18] Dudukovic N. M. Preston A. R. Archie J. J. Glover G. H. Wagner A. D. (2011). High-resolution fMRI reveals match enhancement and attentional modulation in the human medial temporal lobe. *J. Cogn. Neurosci.* 23 670–682 10.1162/jocn.2010.21509 20433244 PMC5746189

[B19] Duncan K. Ketz N. Inati S. J. Davachi L. (2012). Evidence for area CA1 as a match/mismatch detector: a high-resolution fMRI study of the human hippocampus. *Hippocampus* 22 389–398 10.1002/hipo.20933 21484934 PMC3529001

[B20] Duvernoy H. M. (2005). *The Human Hippocampus*, 3rd Edn. Berlin, Heidelberg: Springer-Verlag

[B21] Ekstrom A. D. Bazih A. J. Suthana N. A. Al-Hakim R. Ogura K. Zeineh M. (2009). Advances in high-resolution imaging and computational unfolding of the human hippocampus. *Neuroimage* 47 42–49 10.1016/j.neuroimage.2009.03.017 19303448 PMC2689320

[B22] Eldridge L. L. Engel S. A. Zeineh M. M. Bookheimer S. Y. Knowlton B. J. (2005). A dissociation of encoding and retrieval processes in the human hippocampus. *J. Neurosci.* 25 3280–3286 10.1523/JNEUROSCI.3420-04.2005 15800182 PMC6724896

[B23] Fanselow M. S. Dong H.-W. (2010). Are the dorsal and ventral hippocampus functionally distinct structures? *Neuron* 65 7–19 10.1016/j.neuron.2009.11.031 20152109 PMC2822727

[B24] Fries P. (2009). The model- and the data-gamma. *Neuron* 64 601–602 10.1016/j.neuron.2009.11.024 20005817

[B25] Grill-Spector K. Henson R. Martin A. (2006). Repetition and the brain: neural models of stimulus-specific effects. *Trends Cogn. Sci.* 10 14–23 10.1016/j.tics.2005.11.006 16321563

[B26] Grossberg S. (1971). Pavlovian pattern learning by nonlinear neural networks. *Proc. Natl. Acad. Sci. U.S.A.* 68 828–831 10.1073/pnas.68.4.828 4323791 PMC389053

[B27] Guzowski J. F. Knierim J. J. Moser E. I. (2004). Ensemble dynamics of hippocampal regions CA3 and CA1. *Neuron* 44 581–584 10.1016/j.neuron.2004.11.003 15541306

[B28] Hunsaker M. R. Kesner R. P. (2013). The operation of pattern separation and pattern completion processes associated with different attributes or domains of memory. *Neurosci. Biobehav. Rev.* 37 36–58 10.1016/j.neubiorev.2012.09.014 23043857

[B29] Jensen O. Lisman J. E. (1996). Hippocampal CA3 region predicts memory sequences: accounting for the phase precession of place cells. *Learn. Mem*.** 3 279–287 10.1101/lm.3.2-3.279 10456097

[B30] Kesner R. P. Hopkins R. O. (2006). Mnemonic functions of the hippocampus: a comparison between animals and humans. *Biol. Psychol.* 73 3–18 10.1016/j.biopsycho.2006.01.004 16473455

[B31] Kesner R. P. Hunsaker M. R. Warthen M. W. (2008). The CA3 subregion of the hippocampus is critical for episodic memory processing by means of relational encoding in rats. *Behav. Neurosci.* 122 1217–1225 10.1037/a0013592 19045941

[B32] Lacy J. W. Yassa M. A. Stark S. M. Muftuler L. T Stark C. E. L. (2011). Distinct pattern separation related transfer functions in human CA3/dentate and CA1 revealed using high-resolution fMRI and variable mnemonic similarity. *Learn. Mem*.** 18 15–18 10.1101/lm.1971111 21164173 PMC3023966

[B33] Lee I. Hunsaker M. R. Kesner R. P. (2005). The role of hippocampal subregions in detecting spatial novelty. *Behav. Neurosci.* 119 145–153 10.1037/0735-7044.119.1.145 15727520

[B34] Lee I. Kesner R. P. (2004a). Encoding versus retrieval of spatial memory: double dissociation between the dentate gyrus and the perforant path inputs into CA3 in the dorsal hippocampus. *Hippocampus* 14 66–76 10.1002/hipo.10167 15058484

[B35] Lee I. Rao G. Knierim J. J. (2004b). A double dissociation between hippocampal subfields: differential time course of CA3 and CA1 place cells for processing changed environments. *Neuron* 42 803–815 10.1016/j.neuron.2004.05.010 15182719

[B36] Libby L. A. Ekstrom A. D. Ragland J. D. Ranganath C. (2012). Differential connectivity of perirhinal and parahippocampal cortices within human hippocampal subregions revealed by high-resolution functional imaging. *J. Neurosci.* 32 6550–6560 10.1523/JNEUROSCI.3711-11.2012 22573677 PMC3374643

[B37] Lisman J. E. Grace A. A. (2005). The hippocampal-VTA loop: controlling the entry of information into long-term memory. *Neuron* 46 703–713 10.1016/j.neuron.2005.05.002 15924857

[B38] Logothetis N. K. (2008). What we can do and what we cannot do with fMRI. *Nature* 453 869–878 10.1038/nature06976 18548064

[B39] Lörincz A Buzsáki G. (2000). Two-phase computational model training long-term memories in the entorhinal-hippocampal region. *Ann. N. Y. Acad. Sci.* 911 83–111 10.1111/j.1749-6632.2000.tb06721.x 10911869

[B40] Ludowig E. Trautner P. Kurthen M. Schaller C. Bien C. G. Elger C. E. (2008). Intracranially recorded memory-related potentials reveal higher posterior than anterior hippocampal involvement in verbal encoding and retrieval. *J. Cogn. Neurosci.* 20 841–851 10.1162/jocn.2008.20507 18201126

[B41] Malykhin N. V. Lebel R. M. Coupland N. J. Wilman A. H. Carter R. (2010). In vivo quantification of hippocampal subfields using 4.7 T fast spin echo imaging. *Neuroimage* 49 1224–1230 10.1016/j.neuroimage.2009.09.042 19786104

[B42] Marr D. (1971). Simple memory: a theory for archicortex. *Philos. Trans. R. Soc. Lond. B Biol. Sci*.** 262 23–81 10.1098/rstb.1971.0078 4399412

[B43] McClelland J. L. Goddard N. H. (1996). Considerations arising from a complementary learning systems perspective on hippocampus and neocortex. *Hippocampus* 6 654–665 10.1002/(SICI)1098-1063(1996)6:6<654::AID-HIPO8>3.0.CO;2-G 9034852

[B44] McClelland J. L. Rumelhart D. E. (1985). Distributed memory and the representation of general and specific information. *J. Exp. Psychol. Gen.* 114 159–197 10.1037/0096-3445.114.2.159 3159828

[B45] McNaughton B. Morris R. (1987). Hippocampal synaptic enhancement and information storage within a distributed memory system. *Trends Neurosci.* 10 408–415 10.1016/0166-2236(87)90011-7

[B46] Moser M. B. Moser E. I. (1998). Functional differentiation in the hippocampus. *Hippocampus* 8 608–619 10.1002/(SICI)1098-1063(1998)8:6<608::AID-HIPO3>3.0.CO;2-7 9882018

[B47] Mueller S. G. Schuff N. Yaffe K. Madison C. Miller B. Weiner M. W. (2010). Hippocampal atrophy patterns in mild cognitive impairment and Alzheimer’s disease. *Hum. Brain Mapp*.** 31 1339–1347 10.1002/hbm.20934 20839293 PMC2943433

[B48] Mueller S. Stables L. Du A. Schuff N. Truran D. Cashdollar N. (2007). Measurement of hippocampal subfields and age-related changes with high resolution MRI at 4T. *Neurobiol. Aging* 28 719–726 10.1016/j.neurobiolaging.2006.03.007 16713659 PMC1820772

[B49] Newmark R. E. Schon K. Ross R. S. Stern C. E. (2013). Contributions of the hippocampal subfields and entorhinal cortex to disambiguation during working memory. *Hippocampus* 23 467–475 10.1002/hipo.22106 23504938 PMC4419744

[B50] Norman K. A O’Reilly R. C. (2003). Modeling hippocampal and neocortical contributions to recognition memory: a complementary-learning-systems approach. *Psychol. Rev.* 110 611–646 10.1037/0033-295X.110.4.611 14599236

[B51] Ojemann J. G. Akbudak E. Snyder A. Z. McKinstry R. C. Raichle M. E. Conturo T. E. (1997). Anatomic localization and quantitative analysis of gradient refocused echo-planar fMRI susceptibility artifacts. *Neuroimage* 6 156–167 10.1006/nimg.1997.0289 9344820

[B52] Olsen R. K. Nichols E. A. Chen J. Hunt J. F. Glover G. H. Gabrieli J. D. E., et al. (2009). Performance-related sustained and anticipatory activity in human medial temporal lobe during delayed match-to-sample. *J. Neurosci.* 29 11880–11890 10.1523/JNEUROSCI.2245-09.2009 19776274 PMC2775810

[B53] O’Reilly R. C. McClelland J. L. (1994). Hippocampal conjunctive encoding, storage, and recall: avoiding a trade-off. *Hippocampus* 4 661–682 10.1002/hipo.450040605 7704110

[B54] O’Reilly R. C. Rudy J. W. (2001). Conjunctive representations in learning and memory: principles of cortical and hippocampal function. *Psychol. Rev.* 108 311–345 10.1037/0033-295X.108.2.311 11381832

[B55] Petersen R. C. (2004). Mild cognitive impairment as a diagnostic entity. *J. Intern. Med*. 256 183–94 10.1111/j.1365-2796.2004.01388.x 15324362

[B56] Petersen R. C. Smith G. E. Waring S. C. Ivnik R. J. Tangalos E. G. Kokmen E. (1999). Mild cognitive impairment: clinical characterization and outcome. *Arch. Neurol.* 56 303–308 10.1001/archneur.56.3.303 10190820

[B57] Poppenk J. Evensmoen H. R. Moscovitch M. Nadel L. (2013). Long-axis specialization of the human hippocampus. *Trends Cogn. Sci.* 17 230–240 10.1016/j.tics.2013.03.005 23597720

[B58] Schacter D. L. Wagner A. D. (1999). Medial temporal lobe activations in fMRI and PET studies of episodic encoding and retrieval. *Hippocampus* 9 7–24 10.1002/(SICI)1098-1063(1999)9:1<7::AID-HIPO2>3.0.CO;2-K 10088896

[B59] Schapiro A. C. Kustner L. V. Turk-Browne N. B. (2012). Shaping of object representations in the human medial temporal lobe based on temporal regularities. *Curr. Biol.* 22 1622–1627 10.1016/j.cub.2012.06.056 22885059 PMC3443305

[B60] Scoville W. B. Milner B. (1957). Loss of recent memory after bilateral hippocampal lesions. *J. Neuropsychiatry Clin.**Neurosci*. 12 103–113 10.1136/jnnp.20.1.11 10678523

[B61] Small S. A. Schobel S. A. Buxton R. B. Witter M. P. Barnes C. A. (2011). A pathophysiological framework of hippocampal dysfunction in ageing and disease. *Nat. Rev. Neurosci.* 12 585–601 10.1038/nrn3085 21897434 PMC3312472

[B62] Smith S. M. Beckmann C. F. Andersson J. Auerbach E. J. Bijsterbosch J. Douaud G. (2013). Resting-state fMRI in the human connectome project. *Neuroimage* 15 144–168 10.1016/j.neuroimage.2013.05.039 23702415 PMC3720828

[B63] Suthana N. Ekstrom A. Moshirvaziri S. Knowlton B. Bookheimer S. (2011). Dissociations within human hippocampal subregions during encoding and retrieval of spatial information. *Hippocampus* 21 694–701 10.1002/hipo.20833 20882543 PMC3026858

[B64] Suthana N. Fried I. (2012). Percepts to recollections: insights from single neuron recordings in the human brain. *Trends Cogn. Sci.* 16 427–436 10.1016/j.tics.2012.06.006 22795560 PMC5378553

[B65] Treves A. Rolls E. T. (1992). Computational constraints suggest the need for two distinct input systems to the hippocampal CA3 network. *Hippocampus* 2 189–199 10.1002/hipo.450020209 1308182

[B66] van Strien N. M. Cappaert N. L. M. Witter M. P. (2009). The anatomy of memory: an interactive overview of the parahippocampal–hippocampal network. *Nat. Rev. Neurosci.* 10 272–282 10.1038/nrn2614 19300446

[B67] Vazdarjanova A. Guzowski J. F. (2004). Differences in hippocampal neuronal population responses to modifications of an environmental context: evidence for distinct, yet complementary, functions of CA3 and CA1 ensembles. *J. Neurosci.* 24 6489–6496 10.1523/JNEUROSCI.0350-04.2004 15269259 PMC6729865

[B68] Weiskopf N. Hutton C. Josephs O. Deichmann R. (2006). Optimal EPI parameters for reduction of susceptibility-induced BOLD sensitivity losses: a whole-brain analysis at 3 T and 1.5 T. *Neuroimage * 33 493–504 10.1016/j.neuroimage.2006.07.029 16959495

[B69] Wills T. J. Lever C. Cacucci F. Burgess N O’Keefe J. (2005). Attractor dynamics in the hippocampal representation of the local environment. *Science* 308 873–876 10.1126/science.1108905 15879220 PMC2680068

[B70] Wilson I. A. Ikonen S. Gallagher M. Eichenbaum H. Tanila H. (2005). Age-associated alterations of hippocampal place cells are subregion specific. *J. Neurosci.* 25 6877–6886 10.1523/JNEUROSCI.1744-05.2005 16033897 PMC6725350

[B71] Wisse L. E. M. Gerritsen L. Zwanenburg J. J. M. Kuijf H. J. Luijten P. R. Biessels G. J. (2012). Subfields of the hippocampal formation at 7 T MRI: in vivo volumetric assessment. *Neuroimage* 61 1043–1049 10.1016/j.neuroimage.2012.03.023 22440643

[B72] Yassa M. A. Mattfeld A. T. Stark S. M Stark C. E. L. (2011). Age-related memory deficits linked to circuit-specific disruptions in the hippocampus. *Proc. Natl. Acad. Sci. U.S.A.* 108 8873–8878 10.1073/pnas.1101567108 21555581 PMC3102362

[B73] Yassa M. A. Muftuler L. T Stark C. E. L. (2010a). Ultrahigh-resolution microstructural diffusion tensor imaging reveals perforant path degradation in aged humans in vivo. *Proc. Natl. Acad. Sci. U.S.A.* 107 12687–12691 10.1073/pnas.1002113107 20616040 PMC2906542

[B74] Yassa M. A. Stark S. M. Bakker A. Albert M. S. Gallagher M. Stark C. E. (2010b). High-resolution structural and functional MRI of hippocampal CA3 and dentate gyrus in patients with amnestic mild cognitive impairment. *Neuroimage* 51 1242–1252 10.1016/j.neuroimage.2010.03.040 20338246 PMC2909476

[B75] Yassa M. A Stark C. E. L. (2009). A quantitative evaluation of cross-participant registration techniques for MRI studies of the medial temporal lobe. *Neuroimage* 44 319–327 10.1016/j.neuroimage.2008.09.016 18929669

[B76] Zeineh M. M. Engel S. A. Thompson P. M. Bookheimer S. Y. (2003). Dynamics of the hippocampus during encoding and retrieval of face-name pairs. *Science* 299 577–580 10.1126/science.1077775 12543980

